# Critical Function of PRDM2 in the Neoplastic Growth of Testicular Germ Cell Tumors

**DOI:** 10.3390/biology5040054

**Published:** 2016-12-14

**Authors:** Erika Di Zazzo, Carola Porcile, Silvia Bartollino, Bruno Moncharmont

**Affiliations:** Department of Health Sciences “*Vincenzo Tiberio*”, University of Molise, Campobasso 86100, Italy; carola.porcile@unimol.it (C.P.); silvia.bartollino@unimol.it (S.B.); moncharmont@unimol.it (B.M.)

**Keywords:** *PRDM* gene family, RIZ1, cancer, testicular germ cell tumors, cell proliferation

## Abstract

Testicular germ cell tumors (TGCTs) derive from primordial germ cells. Their maturation is blocked at different stages, reflecting histological tumor subtypes. A common genetic alteration in TGCT is a deletion of the chromosome 1 short arm, where the *PRDM2* gene, belonging to the *P*ositive *R*egulatory domain gene (*PRDM*) family, is located. Expression of *PRDM2* gene is shifted in different human tumors, where the expression of the two principal protein forms coded by *PRDM2* gene, RIZ1 and RIZ2, is frequently unbalanced. Therefore, *PRDM2* is actually considered a candidate tumor suppressor gene in different types of cancer. Although recent studies have demonstrated that *PRDM* gene family members have a pivotal role during the early stages of testicular development, no information are actually available on the involvement of these genes in TGCTs. In this article we show by qRT-PCR analysis that *PRDM2* expression level is modulated by proliferation and differentiation agents such as estradiol, whose exposure during fetal life is probably an important risk factor for TGCTs development in adulthood. Furthermore in normal and cancer germ cell lines, PRDM2 binds estradiol receptor α (ERα) and influences proliferation, survival and apoptosis, as previously reported using MCF-7 breast cancer cell line, suggesting a potential tumor-suppressor role in TGCT formation.

## 1. Introduction

Testicular germ cell tumors (TGCTs) are the most common malignant tumors in young males, representing the major cause of cancer death in 15 to 34 years old males. Ninety-five percent of TGCTs originate from germ cells and are classified into seminoma and non-seminoma germ cell tumors (NSGCTs), including embryonic cell carcinoma, choriocarcinoma, yolk sac tumors and teratoma. Tumors with diverse cell components, e.g., seminoma and embryonic cell carcinoma, are generally indicated as mixed germ cell tumors. Seminomas and NSGCTs also show peculiar clinical features and significant differences in therapy and prognosis [1,2,3,4].

In the last four decades, the incidence of TGCT doubled. Despite the efficacy of primary therapy, the discovery of alternative adjuvant therapies aimed to limit relapses and prevent drug resistance remains a priority. The molecular mechanisms underlying the onset, development and progression of seminomas have not been explained yet. The genetic aberrations causing TGCT are complex; in fact, the development of seminomas involves triploid/tetraploid chromosomes, short arm amplification of chromosome 12, inducing *CCND2* gene (cyclin D2) hyper-expression [5] and deletions of chromosomes 1, 3 or 11 short arms [4]. Whereas the etiology of TGCTs remains undefined, some authors described a possible hormone-dependency of TGCT and formulated a hypothesis about a defect in the estrogen signaling mechanism [6,7,8,9]. For example, *in utero* the exposure to particular hormones (e.g., estrogens) during testis differentiation represents a risk factor for subsequent tumor development [10]. In addition, it was proposed that estrogen’s action on testicular cell transformation might involve oxidative DNA damage mediated through estrogen receptors [11].

Estrogen signaling is exerted by two members of the nuclear receptors superfamily, estrogen receptor α (ERα) and β (ERβ). They regulate transcription in a hormone-dependent manner. These receptors, activated by estradiol binding, associate with other co-activators and repressors and control the expression of target genes [12,13].

In the 1p chromosomal region, frequently deleted in TGCT, is located the *PRDM2*/*RIZ* gene [14,15], suggesting a TGCT-suppressor role. The RIZ protein is involved in the alteration of the estrogen transduction pathway through its hormone-dependent interaction with estrogen receptors [16,17,18]. Moreover, it localizes on estrogen-regulated gene promoters, acting as a co-activator when its methyltransferase activity is inhibited by estradiol [16].

The human *PRDM* family gene contains 17 members that encode for proteins characterized by a PR/SET domain and a different number of zinc-finger domains, aimed to regulate gene expression [19,20,21,22]. Generally there are two alternative forms of PRDM proteins, PR *plus* (PR+) forms and PR *minus* (PR−) forms, of which one differs from the other one only for the lack of the PR domain. The *PRDM2* gene encodes for two major proteins: RIZ1 (PR+) and RIZ2 (PR−) [23]. These two alternative products are involved in tumorigenesis with an unusual *yin-yang* manner. A large number of human cancers, including breast, liver, bone, lung, colon, neuroendocrine cancers and melanoma are characterized by the loss or the decreased expression of the PR+ form and a normal or upregulated expression of PR− form [24,25,26,27,28,29,30,31]. RIZ gene deletion occurs also in colon, breast and liver cancers [5,32,33]. No data are actually available about the expression and the role of *PRDM*-family genes in TGCT. In particular, no evidence is available about *PRDM2* gene, whose expression is altered in a number of human hormone-dependent tumors [34]. Moreover, involvement of the *PRDM2* gene products in estrogen activity is still not well characterized in germinal cells.

**Figure A1:**
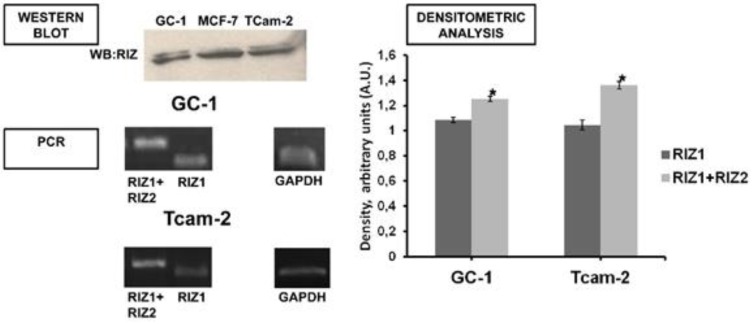
RIZ expression in GC-1 and TCam-2 cell lines. Western blot analyses of total protein cell extracts from GC-1 and TCam-2 cell lines with RIZ1 antibody. MCF-7 cell extract was used as a control. Electrophoresis analysis of amplicons obtained by RT-PCR of cDNA from GC-1 and TCam-2 cells in basal condition (6% FBS DMEM for GC-1 cells and 10% FCS RPMI 1640 for TCam-2 cells). Densitometric analysis of the gels derived from three independent experiments, normalized for GAPDH signals.

To better clarify these aspects, we analyzed RIZ expression levels and its modulation by estrogens using as a model the normal mouse spermatogonial GC-1 and the seminoma TCam-2 cell lines, because both of them express RIZ proteins (RT-PCR analysis, densitometric analysis and Western blot analysis are reported in Appendix Figure A1). Furthermore we studied the RIZ proteins potential role into the mechanism responsible for tumorigenesis.

## 2. Materials and Methods

### 2.1. Cell Culture

GC-1 cell line from American Type Culture Collection (ATCC, Manassas, VA, USA) was maintained in DMEM (Invitrogen, Carlsbad, CA, USA) supplemented with 6% fetal bovine serum, FBS (Invitrogen). TCam-2 cell line (kindly provided by Prof. L. H. Looijenga, Erasmus MC-University Medical Center Rotterdam, Rotterdam, The Netherlands and P. Chieffi, University of Campania “Luigi Vanvitelli”, Caserta, Italy) was cultured in RPMI 1640 (Invitrogen) containing 10% fetal calf serum, FCS. Cell cultures were maintained in humidified 95% air and 5% CO_2_ atmosphere at 37 °C. For treatments, 70% confluent cells were cultured in DMEM without phenol red and serum for one day and subsequently treated for 24 hours with 100 nM 17β-estradiol (E2), 10 nM 5α-dihydrotestosterone (DHT), 10 nM insulin-like growth factor (IGF-1) and 10 nM retinoic acid (RA) (Sigma-Aldrich Co., St. Louis, MO, USA).

### 2.2. Plasmid Transient Transfection

Plasmids carrying RIZ1 or RIZ2 coding sequences were obtained as previously described [35]. An empty vector (pSG5) was transfected in the control sample. Plasmid transfection was achieved with Lipofectamine^®^ reagent (GIBCO BRL, Life Technologies, Rockville, MD, USA) according to manufacturer’s instructions. In each experiment, the plasmid pEGFPC3 was co-transfected to detect and adapt the transfection efficiency by microscopy analysis. All presented data derive from experiments with transfection efficiency greater than 55% and a variation below 20%.

### 2.3. RNA Isolation and Quantitative Reverse-Transcription PCR (qRT-PCR) Analysis

Purification of GC-1 and TCam-2 cells total RNA (1 µg) was achieved with TRIzma Reagent (Sigma-Aldrich Co.) following manufacturer’s instructions. RNA samples were eluted in 50 μL of water treated with diethylpyrocarbonate and stored at −80 °C. The quality of RNA was assessed by gel electrophoresis in denaturing conditions and by evaluation of 260/280 nm and 260/230 nm absorbance ratios: RNA samples with absorbance ratio 260/280 nm lower than 1.9 or with absorbance ratio 260/230 lower than 2.2 were discarded. RNA samples were then treated with 40 U of RNAse-free DNAse-I (Boehringer Mannheim, Indianapolis, IN, USA) for 45 minutes at 37 °C. To exclude the presence of genomic DNA, PCR amplification was performed on RNA samples not reverse-transcribed, too. MMLV-Reverse Transcriptase and random primers from Bio-Rad Laboratories Inc. (Hercules, CA, USA) were used to reverse-transcribe total RNA.

cDNA aliquots were analyzed by qRT-PCR with the SYBR Green PCR Master Mix (Bio-Rad Laboratories Inc., Hercules, CA, USA) in a Mastercycler ep Realplex (Eppendorf, Milan, Italy). Relative mRNA expression was determined by the ΔΔ-Ct method [36] using GAPDH mRNA expression levels as endogenous control. Primer sets used are reported in Supplementary Material. Serial cDNA dilutions were analyzed to ensure the linearity of the PCR reaction and to evaluate its efficiency. cDNA samples were amplified in triplicate and the melting curves were analyzed to verify the specificity of reaction.

### 2.4. Western Blot and Immunoprecipitation

Subconfluent GC-1 or TCam-2 cells were treated with 100 nM 17β-Estradiol (E2) (Sigma-Aldrich) at different time intervals (0–5–15–30–60 minutes). Electrophoresis and Western blot analysis were performed as described elsewhere [37] by using the rabbit polyclonal anti-RIZ antibodies (ab9710, Abcam Ltd., Cambridge, UK) or the rabbit polyclonal anti-ERα antibodies (Santa Cruz Biotechnology Inc., Santa Cruz, CA, USA). The same membrane was stripped and reprobed with the mouse monoclonal anti-α-tubulin antibody (Sigma-Aldrich). For immunoprecipitation, 1 mg of total protein cell lysate was incubated with 4 µg of rabbit polyclonal antibodies RIZ N-20 (Santa Cruz Biotechnology Inc.) specific for RIZ. The clones of the antibodies used are reported in Supplementary Material. Immunocomplexes were pulled down with anti-rabbit IgG beads (ExactaCruz, Santa Cruz Biotechnology Inc.), according to the manufacturer’s instructions, and upon recovery subjected to Western blot analysis. Densitometric analysis was performed with ImageJ software (ImageJ, U.S. National Institutes of Health, Bethesda, Maryland, USA, http://imagej.nih.gov/ij/) using the “Gel Plot” plug-in.

### 2.5. Cell Growth Analysis

Cell proliferation was evaluated by cell counting and by MTT assay (Sigma-Aldrich Co.) as previously indicated [38].

### 2.6. Clonogenic Assay

Clonogenic assay was performed as described elsewhere [39]. Transfected GC-1 cells (3 × 10^2^) expressing RIZ1, RIZ2 or empty vector (pSG5) were resuspended in DMEM, seeded into 6 well plates and cultured for 15 days. Clones were fixed at room temperature with a solution containing 0.5% crystal violet/6% glutaraldehyde (Sigma-Aldrich) for 30 minutes. Clones were counted with ImageJ software using the “analyze particles” routine.

### 2.7. BrdU Incorporation Assay

DNA synthesis was assessed using the 5-Bromo-2′-deoxy-uridine labelling and detection kit (Roche Applied Science, Penzberg, Germany), as previously described [38]. GC-1 cells were transfected and after 24 hours, were plated in 96-well plates (4.0 × 10^3^ cell/well) and cultured for an additional 24–48 hours; 10 µM BrdU (Roche Applied Science) was added in the last 4 hours and subsequently the cell lysate was processed following the manufacturer’s instructions.

### 2.8. TUNEL Assay

Apoptosis was assessed by APO-BrdU TUNEL assay detection kit, following manufacturer’s instructions (Becton Dickinson, Franklin Lakes, NJ, USA). Cells were analyzed by flow cytometer (FACS-CANTO II Becton–Dickinson).

### 2.9. Statistical Analysis

All data are presented as the means ± SD of at least three experiments in triplicates. Statistical significance between groups was evaluated using Student’s t-test. All statistical analyses were conducted using JMP Software purchased by Statistical Discovery SAS Institute (*p* < 0.05, statistical significance; *p* < 0.001, high statistical significance).

## 3. Results

### 3.1. PRDM2 Expression Level is Modulated by Spermatogonial Proliferation and Differentiation Agents

**Figure 1:**
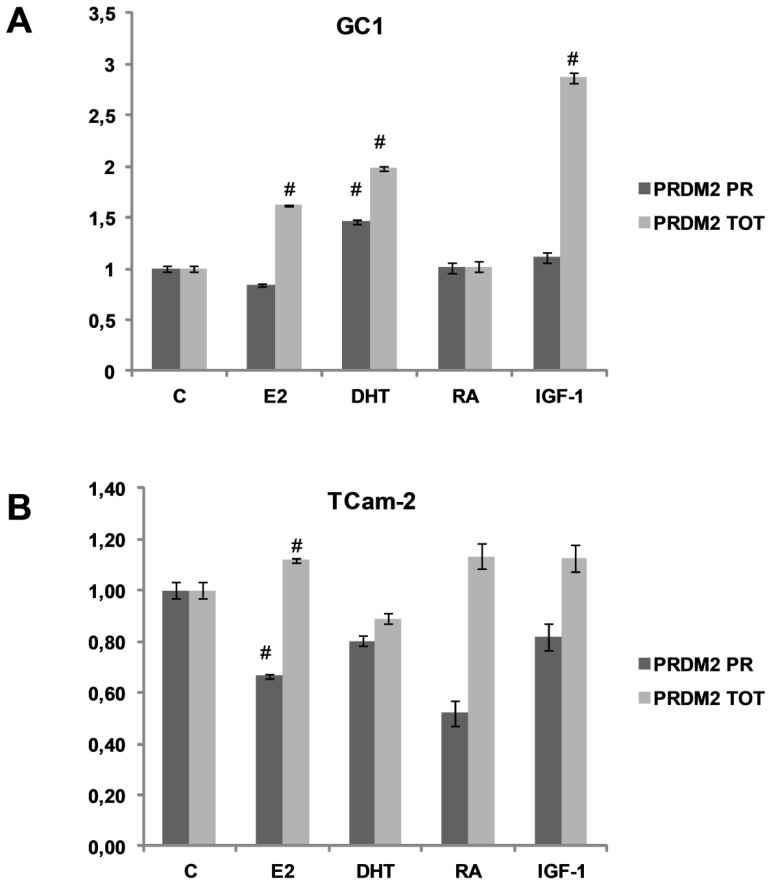
Modulation of *PRDM2* gene expression by proliferation and differentiation agents. The transcripts encoded by *PRDM2* gene was measured by qRT-PCR after a treatment with 100 nM E2, 10 nM DHT, 10 nM retinoic acid (RA) or 10 nM insulin-like growth factor (IGF-1) for 48 hours. The PRDM2 PR and PRDM2 TOT sets of primers recognize sequences on the region coding PR domain of RIZ1 or on a region common to both RIZ1 and RIZ2 that encodes a sequence near the C-terminal end, respectively. The expression level is indicated as fold changes from basal conditions. Histograms represent the averages (+/− standard error) from at least three independent experiments, normalized for the expression of the control housekeeping gene GAPDH (# indicates *p* < 0.05 for RIZ1 or total RIZ, respectively, versus their untreated control). (**A**) qRT-PCR of total RNA/cDNA extracted from GC-1 cell line; (**B**) qRT-PCR of mRNA/cDNA extracted from TCam-2 cell line.

Spermatogonial proliferation and differentiation agents modulation of *PRDM2* gene transcription was analyzed by qRT-PCR, using two sets of primers, PRDM2 PR and PRDM2 TOT, that recognize sequences on the region coding PR domain of RIZ1 or on a region coding a sequence near the C-terminal common to both RIZ1 and RIZ2, respectively [35]. In both GC-1 mouse spermatogonial normal cell line and in TCam-2 human spermatogonial cancer cell line (Figure 1, panel A or B, respectively), treatment with proliferation-inducing agents modulated the relative ratio between the major transcripts coded by *PRDM2* gene. In GC-1 cell line, E2 or IGF-1 treatment induced a significant increase of RIZ2 transcript. DHT treatment modulated positively both *PRDM2* forms, as previously observed in EPN cells (normal ephitelium prostate cell line) [34]. In both cell types, E2 increased the expression level of the RIZ forms lacking the PR domain. In addition, in TCam-2 cells E2 reduced RIZ1 expression levels. IGF-1 treatment increased expression levels of the RIZ forms lacking the PR domain in GC-1 cells. In conclusion, RIZ was still expressed in tumor phenotype and, according with previous results obtained in MCF-7 breast cancer cell line, E2 modifies the RIZ1/RIZ2 ratio, lowering the expression level of RIZ1 and raising the RIZ2 one [35,37]. E2 treatment also induces GC-1 [40] and TCam-2 proliferation (data not shown) [41] suggesting that the effect on cell growth is exerted by RIZ proteins. Taken together, *PRDM2* expression was maintained in cancer cell lines, suggesting that probably in tumors the mechanism of action of RIZ proteins is modified.

### 3.2. In GC-1 and TCam-2 Cell Lines RIZ1 Binds ERα and This Interaction is Modulated by Estradiol Treatment

**Figure 2:**
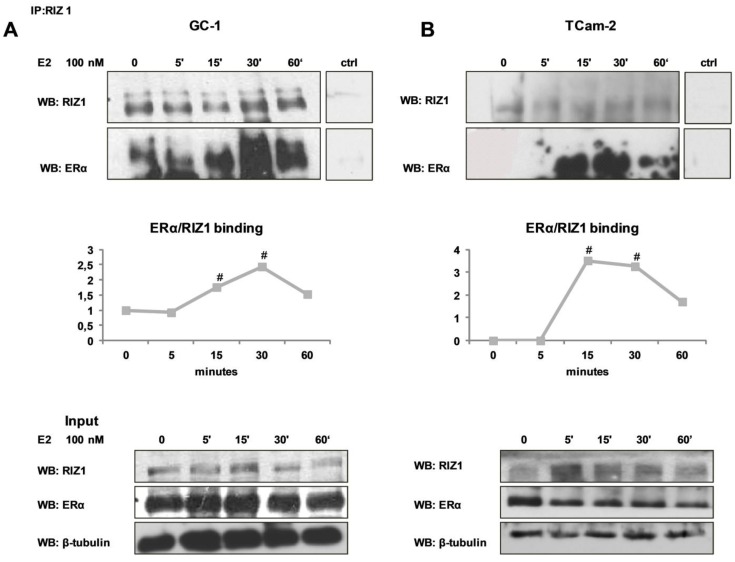
RIZ1/ERα interaction. The interaction between RIZ1 and ERα was evaluated by immunoprecipitation, SDS-PAGE and Western blot analysis of total protein cell extracts from GC-1 and TCam-2 cell lines treated with 100 nM estradiol (E2) for 5, 15, 30 or 60 minutes. Immunoprecipitation was performed with N-20 anti RIZ1 antibody; Western blot was performed with ab9710 anti-RIZ1, anti-ERα or anti-α-tubulin antibodies. An anti-rabbit IgG beads were used in the absence of N-20 anti RIZ1 antibody as a control (ctrl). Input: total protein cell extracts. ER/RIZ1 binding graphs represent the ratio between the value obtained by densitometric analysis of the bands corresponding to ERα and RIZ1 by ImageJ software. The blots are representative of three independent experiments (# indicates *p* < 0.05). (**A**) GC-1 cell line; (**B**) TCam-2 cell line.

It has been previously demonstrated that RIZ1 binds ERα [16]. To investigate whether RIZ1 binds ERα in normal GC-1 cell line and in TCam2 cancer cell line, following E2 treatment total protein extract was immunoprecipitated with polyclonal anti-RIZ1 antibodies. Co-immunoprecipitated proteins were pulled down and then processed and analyzed by SDS-PAGE followed by Western blot using antibodies against RIZ or ERα proteins (Figure 2). In GC-1 cells RIZ1 was bound to ERα in basal condition. The interaction raised after 15 minutes and peaked after 30 minutes of 100 nM E2 treatment (Figure 2A). In TCam-2 cells RIZ1 was not co-immunoprecipitated with ERα in basal condition and the interaction increased significantly only after 15 minutes of E2 treatment and declined after 60 minutes (Figure 2B). The responsiveness to estradiol treatment was confirmed as control by the rapid increase of ERK1/ERK2 phosphorylation (data not shown) [41].

### 3.3. RIZ1 Over-Expression Inhibits GC-1 Cells Proliferation and Survival 

**Figure 3:**
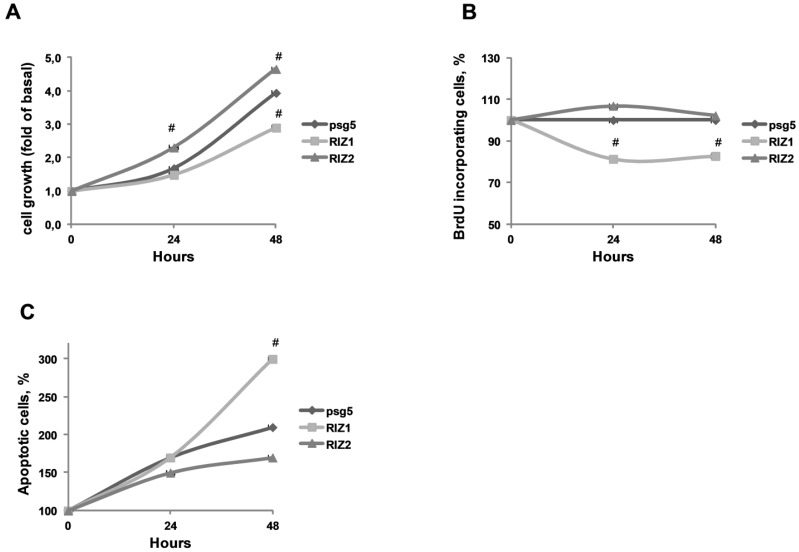
Effect on cell proliferation, survival and apoptosis upon ectopic expression of RIZ1 or RIZ2 in spermatogonial cell line GC-1. (**A**) For the colorimetric MTT assay, GC-1 cells transiently transfected with a plasmid encoding for RIZ1, RIZ2 or the empty vector (pSG5) were plated at the same density and allowed to grow for 0, 24 or 48 hours. MTT was added in the last 2 hours, formazan precipitates were dissolved with dimethyl sulfoxide reagent and absorbance read at 570 nm [38]. Values are the mean (±SE) of four analyses from three independent experiments # *p* < 0.05 vs. control; (**B**) For the BrdU incorporation assay, transiently transfected cells with a plasmid encoding for RIZ1, RIZ2 or for empty vector (pSG5) were seeded 24 hours after transfection into 96-well plates and cultured for further 24–48 hours; during the last 4 hours, cells were treated with 10 µM BrdU. The cells were processed following the manufacturer’s instructions and cell lysates were analyzed by an ELISA kit to measure the amount of incorporated BrdU with specific anti-BrdU antibody peroxidase-conjugate. Data are represented as a percentage of basal values. Values are the mean (±SE) of four analyses from three independent experiments # *p* < 0.05 vs. control; (**C**) GC-1 cells, transiently transfected with a plasmid encoding for RIZ1, RIZ2 or with the control vector, were cultured in 60-mm dishes for 24 or 48 hours. Cells were processed following manufacturer’s instruction and finally analyzed by flow cytometer to determine the percentage of apoptotic cells. The data are the mean of three independent experiments performed in triplicate (*n* = 9) # *p* < 0.05 vs. control.

To investigate the capability of RIZ1 to reduce the growth of spermatogonial cell line GC-1 in vitro, colorimetric MTT assay was performed on GC-1 cells transiently transfected with the recombinant plasmids encoding for RIZ1 or RIZ2. pSG5 empty vector was used as control. Cells were harvested and analysed at time intervals of 0, 24 or 48 hours. Colorimetric MTT assay showed that RIZ1 over-expression inhibited cell proliferation, whereas RIZ2 over-expression had an increasing effect on it (Figure 3A). In addition, RIZ1 over-expression in GC-1 cells suppressed BrdU incorporation of about 20% compared to mock-transfected GC-1 cells. On the other hand RIZ2 over-expression did not significantly suppress BrdU incorporation (Figure 3B). These results suggested that RIZ1 expression was required for DNA synthesis suppression, similar to the recent results obtained in a malignant meningioma cell line [42].

### 3.4. RIZ1 Over-Expression Induces GC-1 Cells Apopotosis

We investigated the influence of RIZ1 forced expression on apoptosis in GC-1 spermatogonial cell line. As shown in Figure 3C, RIZ1 over-expression significantly increased the number of GC-1 apoptotic cells as compared with the control groups.

### 3.5. PRDM2 Influences Tumor Growth 

**Figure 4:**
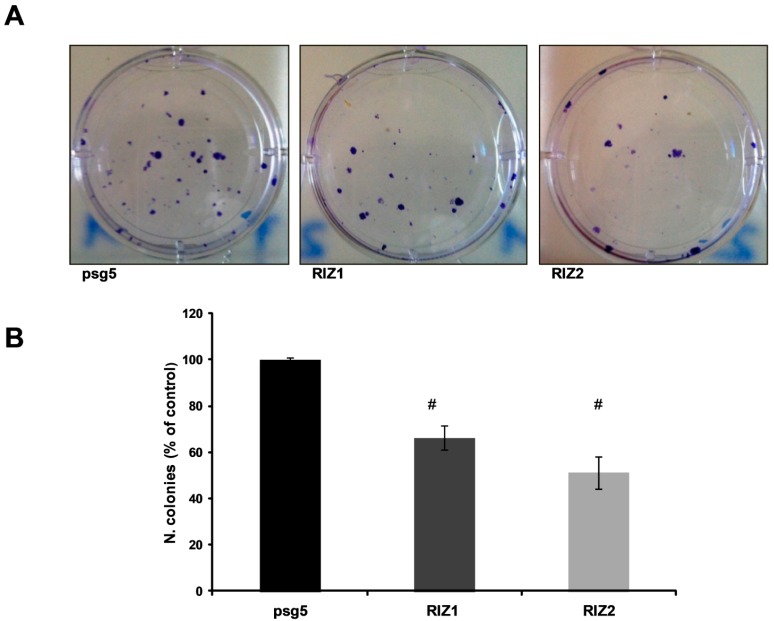
Role of RIZ in GC-1 tumor growth. (**A**) GC-1 cells transiently transfected with indicated vectors, were seeded into 6-well plates. Fifteen days later the clones were fixed at room temperature with a solution containing 0.5% crystal violet/6% glutaraldehyde (Sigma-Aldrich) for 30 minutes. Clones were counted and their cellularity was evaluated by contrast microscopy; (**B**) The histograms represent the averages of colonies from four separate experiments performed in duplicate (*n* = 8). # *p* < 0.05 vs. control.

RIZ1 is silenced in different cancer cell lines and tumors, suggesting that RIZ1 acts as a tumor suppressor. Therefore, we evaluated the effects of ectopic RIZ1 expression on tumor cell clonogenicity. RIZ1 and RIZ2 encoding vectors (or the empty vector as control) were transfected into GC-1 cell line. Three hundred transfected cells were seeded in a 6-well plates and fifteen days later the count of the clones and the evaluation of their morphology by contrast microscopy was performed (Figure 4). Ectopic RIZ1 expression reduced the colony-forming efficiency, as compared to the vector control (40% and 50% respectively). Expression of RIZ1 or RIZ2 was confirmed by qRT-PCR and Western blot analysis [41].

## 4. Discussion

In this report we demonstrated that RIZ1 protein was expressed in spermatogonial-derived cell lines. Consistent with previous results obtained in MCF-7 cell line [35,37], E2 treatment of spermatogonial cells induced a decrease in RIZ1 transcript expression level and a moderate increase of RIZ2 transcript, suggesting a relative increment in RIZ2 expression over RIZ1 (Figure 1).

Several reports suggested that RIZ1 is a downstream effector of estrogen action and that it is involved in regulation of cell proliferation in classical estrogen target tissues [16,18,35]. In our models, RIZ1 binding to ERα, potentiated by estradiol, strongly suggested a pivotal role for RIZ1 in mediating estrogen control of cell growth through ERα.

Genetic data from both animal and human studies demonstrated that RIZ1 has a proven role in cancer etiology. Fourteen different studies show a strong tumor-suppressive activity for RIZ1. For example, in breast, liver and colon cancer RIZ1 over-expression causes G2-M cell cycle arrest and/or apoptosis [43,44,45]. Moreover in many types of human tumors RIZ1 expression is deregulated. However, the RIZ1 functional significance in seminoma development has not been addressed yet. We observed that, when ectopically expressed in GC-1 cells, RIZ1 suppressed the cell survival rate and had a growth-inhibitory effect. These results clearly support the hypothesis that deregulation of RIZ1 could have an essential role in spermatogonial cell transformation. Our data provide important motivation to characterize RIZ1 function in the molecular mechanisms underlying spermatogonial cell transformation. New insights, for example, could arise by using animal tumor models to evaluate the effect of RIZ1 on seminomas in vivo.

Our data indicated also that RIZ1 over-expression induced apoptosis in GC-1 cells as pointed out by the increase in the number of TUNEL-positive cells counted by flow cytometer. Previous reports demonstrated that RIZ1 expression can induce p53 over-expression, and RIZ1 silencing limited p53 expression [42,46]. RIZ1 over-expression modifies antiapoptotic/apoptotic effectors, suggesting that RIZ1 up-regulation could induce cell apoptosis and thus inhibit cell proliferation. Although the mechanism underlying the association between RIZ1 expression and seminomas is still unclear, future studies will focus on the identification of genomic alterations of *PRDM2* gene, whose locus is on chromosome 1p36, on the molecular mechanisms underlying *PRDM2* gene expression modulation and on the cell mechanisms associated with RIZ1 function. Our results strongly suggest that RIZ1 is a promising candidate tumor-suppressor gene in the development of seminomas. We will attempt to discover cell partners interacting with RIZ1 to elucidate how the deregulation of RIZ1 inhibits cell growth. 

## 5. Conclusions

Our findings support further characterization of molecular alterations in TGCT. The understanding of the functional consequences of these alteration in deregulation of tumor suppressor genes in TGCT cells will provide an important approach to discover new diagnostic and therapeutic strategies.

## Supplementary Materials

The following are available online at www.mdpi.com/2079-7737/5/4/54/s1.

## Abbreviations

The following abbreviations are used in this manuscript:

TGCTs	Testicular germ cell tumors
NSGCTs	non-seminoma germ cell tumors
ERα	estrogen receptor α
ERβ	estrogen receptor β
E2	17β-Estradiol
MTT	3-(4,5-dimethylthiazolyl-2)-2,5-diphenyltetrazolium bromide
BrdU	5-bromo-2′-deoxy-uridine
DHT	5α-dihydrotestosterone
SDS-PAGE	Sodium Dodecyl Sulphate-PolyAcrylamide Gel Electrophoresis
