# Supplementary material for: Critical Function of PRDM2 in the Neoplastic Growth of Testicular Germ Cell Tumors
Source: Biology (Basel). 2016 Dec 14;5(4):54. doi: 10.3390/biology5040054 (PMC5192434; doi:10.3390/biology5040054)
Supplement: Supplementary file 1 [file biology-05-00054-s001.docx]

Article

Critical Function of PRDM2 in the Neoplastic Growth of Testicular Germ Cell Tumors

Erika Di Zazzo *, Carola Porcile, Silvia Bartollino and Bruno Moncharmont

**Supplementary Materials**

1. Primer sets fot the amplification of PRDM2 PR, PRDM2 TOT and GAPDH in mouse by qRT-PCR

PRDM2 PR F ACTGGCTCCGCTATGTGAAC

PRDM2 PR R CGCGATTGGCTTTAAGGTT

PRDM2 TOT F CCGGAGAGGGAAGAAGAAAT

PRDM2 TOT R TCATGTTTGCAGAGGTGGAG

GAPDH F TGTGTCCGTCGTGGATCTGA

GAPDH R CCTGCTTCACCACCTTCTTGA

2. Primer sets fot the amplification of PRDM2 PR, PRDM2 TOT and GAPDH in human by qRT-PCR

PRDM2 PR F AATTTGGGATGGATGTGCATTG

PRDM2 PR R GGCGCGATTGGCTTTAAAGT

PRDM2 TOT F CCCCAAGAGCCGGAAAGGGAAGAA

PRDM2 TOT R TCTGGCTCACTTGTCTTCAGTTGT

GAPDH F GGATTTGGTCGTATTGGG

GAPDH R GGAAGATGGTGATGGGATT

3. Clones of antibodies

Anti-RIZ antibody: ab9710, AbCam

Anti-RIZ antibody (N-20): sc-14228, Santa Cruz Biotechnology

Anti-α-Tubulin antibody: T5168, SIGMA

Anti-Erα antibody (C311): sc-787, Santa Cruz Biotechnology

© 2016 by the authors. Submitted for possible open access publication under the
terms and conditions of the Creative Commons Attribution (CC-BY) license (http://creativecommons.org/licenses/by/4.0/).
